# Bayesian random effects modelling with application to childhood anaemia in Malawi

**DOI:** 10.1186/s12889-015-1494-y

**Published:** 2015-02-19

**Authors:** Alfred Ngwira, Lawrence N Kazembe

**Affiliations:** Department of Mathematical Sciences, University of Malawi, Chancellor College, Zomba, Malawi; Department of Statistics and Population Studies, University of Namibia, Windhoek, Namibia

**Keywords:** Binary logistic model, Structured additive, Geo-additive, P-splines

## Abstract

**Background:**

Epidemiological studies in Malawi on child anaemia have neglected the community spatial effect to childhood anaemia. Neglecting the community spatial effect in the model ignores the influence of unobserved or unmeasured contextual variables, and at the same time the resultant model may under estimate model parameter standard errors which can result in erroneous significance of covariates. We aimed at investigating risk factors of childhood anaemia in Malawi with focus on geographical spatial effect.

**Methods:**

We adopted a Bayesian random effect model for child anaemia with district as spatial effect using the 2010 Malawi demographic healthy survey data. We fitted the binary logistic model for the two categories outcome (anaemia (Hb < 11), and no anaemia (Hb ≥ 11)). Continuous covariates were modelled by the penalized splines and spatial effects were smoothed by the two dimensional spline.

**Results:**

Residual spatial patterns reveal Nsanje, Chikhwawa, Salima, Nkhota-kota, Mangochi and Machinga increasing the risk of childhood anaemia. Karonga, Chitipa, Rumphi, Mzimba, Ntchisi, and Chiradzulu reduce the risk of childhood anaemia. Known determinants such as maternal anaemia, child stunting, and child fever, have a positive effect on child anaemia. Furthermore childhood anaemia decreases with child age. It also decreases with wealth index. There is a U relationship between child anaemia and mother age.

**Conclusion:**

Strategies in childhood anaemia control should be tailored to local conditions, taking into account the specific etiology and prevalence of anaemia.

## Background

Childhood anaemia is a global public health problem. According to World Health Organization (WHO) current report on world prevalence of anaemia [1], the global prevalence of anaemia is 24.8% with the highest prevalence in preschool-age children (47.4%). Regional WHO estimates of childhood anaemia shows sub-Saharan Africa (SSA) having the highest prevalence, about 67%, seconded by the South East Asia (65.5%). The latest report though by [2] on world prevalence of anaemia shows that world prevalence of anaemia for preschool-age children has decreased from 47% to 43% and that South Asia, Central and West Africa have the highest prevalence. Malawi, part of the sub-Saharan Africa and in Central Africa has 63% prevalence of childhood anaemia according to the 2010 Malawi Demographic Health Survey (MDHS) report [3]. Consequences of the childhood anaemia are poor cognitive development for mild and moderate anaemia, and death for severe anaemia. Severe anaemia carries a significant risk of death by profound hypoxia and congestive heart failure, or more rarely, by cerebral malaria [4,5].

Epidemiology of childhood anaemia shows multi-factorial risk factors. About 50% of all anaemia cases are due to iron deficiency [6]. Other micronutrients, such as vitamin A, vitamin C, and folate are important in the pathophysiology of anaemia. Infections such as malaria, HIV, bacteraemia caused by organisms such as *Steptococcus pneumoniae, non-typhi Salmonella species, and Haemophilus influenzae type b*, and helminth infections caused by hookworm and *Schistosoma haematobium* are also known to cause anaemia [7,8]. The general mechanisms by which these infections lead to anaemia include blood loss, sequestration of red blood cells by the spleen, haemolysis by antibodies, and anaemia of inflammation (via TNF-alpha and IL-6 production). Previous studies have also shown that socioeconomic factors such as low parental education levels [9], low household incomes, and demographic factors including age, sex [10], and family size [11] affect anaemia. Sickle cell disease has also been recognized as an important risk factor for anaemia in sub-Saharan countries [12].

To our knowledge, studies on childhood anaemia in Malawi have not assessed the geographical heterogeneity in childhood anaemia causes [7,13]. The ignorance of heterogeneity in models according to [14], may lead to biased parameter estimates. But more importantly, geographical heterogeneity can be an effect of unmeasured covariates which may include contextual factors. That is, geographical differences in the causes of anaemia can be partially explained by large-scale variability in environmental drivers, particularly nutritional and infectious causes. Malaria as an infectious cause of anaemia is known to be associated with elevation and land surface temperature. Similarly, nutritional iron deficiency and anaemia-causing helminth infections are known to be associated with the distance to a perennial water body, land surface temperature and the normalized difference vegetation index (NDVI). The environmental drivers of anaemia tend to show a high degree of spatial dependence (i.e. geographical clustering) [15,16]. There are number of studies though outside Malawi [17-21], that have taken into account the geographical heterogeneity in modelling of anaemia, but all these studies have often ignored the flexible approach of using bivariate splines in modelling geographical heterogeneity.

The study of geographical heterogeneity of a health outcome can benefit from the multilevel or spatial mixed model. For example [18,20,21], use a multilevel model and [17,19] use a spatial mixed model. In multilevel models geographical heterogeneity is modelled as a random effect and geographical variation in the outcome variable is assessed via variance partition coefficient (VPC) or intra-class correlation coefficient (ICC). In spatial mixed models, geographical heterogeneity of an outcome is assessed by specifying a spatial correlation structure for individual residuals. A comparison study of a multilevel and a spatial mixed model for investigating place effects on health outcomes by [22] showed a smaller deviance for spatial mixed model than a multilevel model, and that the Moran’s I statistic showed residual spatial autocorrelation unaccounted for by the multilevel model.

Spatial mixed models have been widely used to asses the geographical effect on an outcome ([17,19,23-26], among others). In case of areal data, where individual information for areas is provided, spatial lattice models, which usually consider correlation between adjacent areas of a territory, are considered appropriate. If the data has location coordinates (latitude and longitude or centroids based on the map), then use of a geo-statistical model proves appropriate. In this study for example, there was no individual information for all districts, but districts centroids based on the map could be got. Thus a geo-statistical model either based on kriging or bivariate spline was appropriate [22].

The contribution of this study would be the application of the spatial mixed model in assessing the significance of correlated geographic effect on childhood anaemia which has not been extensively done by assuming the flexible approach of bivariate splines. Furthermore the study would be the first ever to map childhood anaemia in Malawi in terms of residual spatial effects. The map would have important implications for targeting policy as well as the search for left-out variables that might account for these residual spatial patterns.

## Methods

### Study area and data

The study focused on Malawi and used the standard and nationally representative 2010 Malawi Demographic and Health Survey (MDHS) data. The MDHS data was downloaded from the DHS website (http://www.measuredhs.com/login.cfm) after being granted permission. The sampling design was a two stage cluster design with stratification. The primary sampling units were the enumeration areas (EAs), and the secondary sampling units were the households. EAs were stratified in terms of rural and urban. A total of 849 EAs were sampled with 158 in urban areas and 691 in rural areas. A representative total sample of 27345 households was selected for the 2010 MDHS survey. Data collection was by questionnaires. There were three questionnaires, women, men and household questionnaire. Households that were successfully interviewed were 24825, yielding a response rate of 98%. Eligible women that were successfully interviewed were 23020, yielding response rate of 97%. Eligible men that were successfully interviewed were 7175, yielding a response rate of 92%. The data set that was used in this study was child record data set which was based on women and household questionnaire. The child record data set had a total of 19967 children records. The following exclusion criteria based on 2010 MDHS report [3] and MDHS guide to statistics [27] was used to have the final sample for children. Children whose mothers were not listed in the household questionnaire were not included. All children records where haemoglobin level was missing were dropped. The missing covariate values were left unremoved. The final sample size of children was thus 4177.

Data management in terms of extracting and generation of variables from child record data set was done in STATA version 12. Data variables used in this study were based on the variables used in previous studies on childhood anaemia. Response variable in the extracted data set was child anaemia status based on the categorization of child altitude adjusted haemoglobin level. Child anaemia status was a binary variable based on the cut off point of 11Hb. Children whose haemoglobin level was less than 11Hb were taken as anaemic and not anaemic otherwise. The cut off point used in classifying child anaemia into two categories was based on 2010 MDHS report. The covariates in the generated data set were mother education level, family wealth index, child cough, child fever, receiving vitamin A, mother anaemia status, stunting, wasting, underweight, child birth weight, child birth order, house hold size, child age in months, mother age in years, whether child ate meat in previous one month or not, breast feeding in months and district of the child. Child age in months, mother age in years and breast feeding in months were continuous covariates. Stunting, wasting and underweight were based on categorization of height for age, weight for height, and weight for age z-scores respectively using z-score −2 as cut off point. District of the child was labelled *s*_*i*_* ϵ*(1, 2, 3,.., *S*) where the label was corresponding to label on the map.

### Statistical analysis

Univariate logistic regression was performed in STATA statistical software, version 12 to select potential factors of childhood anaemia. Covariates that were associated with anaemia at significance level of 20% were incorporated in the multiple regression models. The significant level of 20% rather than 5% was used in selecting covariates for multiple regression analysis so as to allow more potential covariates to be selected. Two way cross tabulation was then performed in STATA statistical software, version 12 to find percentage distribution of childhood anaemia per district and per covariate categories. Percentages were weighted using the sampling weight to ensure representative sample. The two way cross tabulation with Pearson chi-square (${\mathcal{X}}^2$) test was used to compare groups of categorical variables.

Four multiple logistic models were then fitted using R2BayesX package in software R using child anaemia status as a response. More formally, considering child anaemia status being binary, in this case child anaemia status being distributed as Bernoulli (*p*_*ij*_) where *p*_*ij*_ is the probability of child *j* being anaemic in location *i*, the following models were fitted.

Model 1: $$ \mathrm{logit}\left({p}_{ij}\right)={w}_i^T\gamma $$

Model  2: $$ \mathrm{logit}\left({p}_{ij}\right)={w}_i^T\gamma +{f}_1\left({x}_{i1}\right)+{f}_2\left({x}_{i2}\right)+\dots +{f}_p\left({x}_{ip}\right) $$

Model 3: $$ \mathrm{logit}\left({p}_{ij}\right)={w}_i^T\gamma +{f}_{spat}\left({s}_i\right) $$

Model 4: $$ \mathrm{logit}\left({p}_{ij}\right)={w}_i^T\gamma +{f}_1\left({x}_{i1}\right)+{f}_2\left({x}_{i2}\right)+\dots +{f}_p\left({x}_{ip}\right)+{f}_{spat}\left({s}_i\right) $$

Model 1 was a fixed effects variable model where all variables, categorical and continuous were modelled as fixed effects. In Model 2, categorical variables were modelled as fixed effects and continuous variables were modelled non parametrically by smooth function *f*_*j*_s. In Model 3 all covariates were modelled as fixed effects and district of the child was modelled as a spatial effect. Model 4 was an extension of Model 2 by including a spatial component. In the models, the smooth functions *f*_*j*_ were specified as Bayesian splines. According to [28], this assumes approximating *f*_*j*_ by polynomial splines of degree *l* defined at equally spaced knots ${x}_j^{min}={\zeta}_{j0},{\zeta}_{j1}, \dots, {\zeta}_{js}={x}_j^{max}$ which are within the domain of the covariate *x*_*j*_. The Bayesian spline can be written as a linear combination of *d* = *s* + *l* basis functions, *B*_*m*_, that is,

1 $$ {f}_j\left({x}_j\right)\kern0.5em =\kern0.5em {\displaystyle {\sum}_{m=1}^d{\varepsilon}_{jm}{B}_m\left({x}_j\right)} $$

Now Bayesian estimation of the penalized spline (1) is equivalent in estimating model parameters *ε*_*j*_ = (*ε*_*j*,1_, *ε*_*j*,2_, … , *ε*_*j*,*m*_) where first or second order random walk priors for the regression coefficients are assigned. A first order random walk prior for equidistant knots is given by: *ε*_*j*,*m*_ = *ε*_*j*,*m* − 1_ + *u*_*j*,*m*_ where *m* = 2, 3, …, *d*, and a second order random walk prior for equidistant knots is given by: *ε*_*j*,*m*_ = 2*ε*_*j*,*m* − 1_ + *ε*_*j*,*m* − 2_ + *u*_*j*,*m*_ where *m* = 3, 4, …, *d* and ${u}_{j.m}\sim N\left(0,\ {\tau}_j^2\right)$ are random errors. The spatial effect was modelled by the tensor product of two dimensional spline defined as

2 $$ {f}_{spat}\left({x}_1,{x}_2\right)={\displaystyle {\sum}_i^k{\displaystyle {\sum}_j^k}}{B}_{spat,\ ij}{B}_{1i}\left({x}_1\right){B}_{2j}\left({x}_2\right) $$

where (*x*_1_, *x*_2_) refers to the coordinates of the location of the data point, latitude and longitude, or location centroids based on the map. The prior for *B*_*spat*, *ij*_ = (*B*_*spat*,11_, *B*_*spat*,12_, …, *B*_*spat*,*kk*_) is based on spatial smoothness priors common in spatial statistics (see [29]). The most commonly used prior specification based on the four nearest neighbours is defined as:

$$ {B}_{spat,\ ij}\Big|.\sim N\left({B}_{spat,i-1\ j}+{B}_{spat,i+1, j}+{B}_{spat,i,j-1}+{B}_{spat,i,j+1},\frac{\tau_{ij}^2}{4}\right) $$

for *i*, *j* = 2, …, *k* − 1 with appropriate changes for corners and edges. Since model estimation was by empirical Bayesian method, all variance parameters were treated as unknown constants that were estimated by restricted maximum likelihood (REML) method and hence their priors were not given. The fixed effects were assigned diffuse priors. An advantage of the empirical Bayesian inference over full Bayesian inference is that questions about the convergence of MCMC samples or sensitivity on hyper parameters do not arise [30]. Further more, a comparison of full Bayesian and empirical Bayesian approach in a simulation study, has shown empirical Bayesian approach yielding somewhat better point estimates, especially for Bernoulli distributed responses (see [31]).

## Results

### Descriptive results

Table 1 presents prevalence of childhood anaemia by region. Northern region is generally less anaemic compared to the central and southern region. Districts in the central region with relatively higher prevalence of childhood anaemia are Salima and Nkhota-kota with about 80% and 74% prevalence respectively. In the south, Chikhwawa, Nsanje, Balaka, Neno, Mangochi and Machinga have relatively higher prevalence of childhood anaemia. In the northern region, Nkhata-bay has relatively high prevalence of childhood anaemia with prevalence of about 73%.

**Table 1:** **Prevalence of childhood anaemia by district**

**Region/district**	**%Anaemic (total)**
**Northern region**	**58.71 (728)**
Chitipa	52.42 (189)
Karonga	54.31 (179)
Nkhata-Bay	72.89 (117)
Rumphi	56.78 (113)
Mzimba	59.98 (130)
**Central region**	**64.21 (1,560)**
Kasungu	68.78 (198)
Nkhota-Kota	74.82 (181)
Ntchisi	55.83 (177)
Dowa	65.37 (187)
Salima	80.62 (139)
Lilongwe	58.39 (211)
Mchinji	61.19 (179)
Dedza	65.83 (125)
Ntcheu	60.01 (163)
**Southern region**	**64.11 (1,889)**
Mangochi	73.40 (160)
Machinga	75.24 (141)
Zomba	62.75 (165)
Chiradzulu	44.85 (116)
Blantyre	47.68 (143)
Mwanza	64.83 (121)
Thyolo	54.42 (148)
Mulanje	60.96 (121)
Phalombe	60.65 (190)
Chikwawa	78.52 (150)
Nsanje	72.29 (163)
Balaka	70.39 (139)
Neno	72.76 (132)

Row % of child anaemia by district based on child data.

MDHS 2010 (weighted).

Table 2 shows the burden of childhood anaemia by categorical covariates and group comparison by Pearson chi-square tests. Males have almost the same prevalence of childhood anaemia as females. Also children of rural areas have higher prevalence of childhood anaemia compared to those of the urban. Childhood anaemia prevalence decreases with wealth. Childhood anaemia decrease from no education mothers to secondary education mothers and then increase for the mothers with higher education. Childhood anaemia prevalence increases with cough and fever. Vitamin A is seen as important in reducing childhood anaemia prevalence. Childhood anaemia prevalence also increases with childhood under nutrition. The categorical variables associated with childhood anaemia at 0.05 significance level without controlling for other factors are residence, wealth, mother education, mother anaemia status, underweight, stunting, wasting, cough, fever, and vitamin A. All categorical covariates in Table 2 were included in the multiple logistic models except the house hold size, ate meat, and child birth order number because their Pearson chi-square p-values are more than 0.2.

**Table 2:** **Prevalence of childhood anaemia by categorical covariates and bivariate Pearson chi-square test p-values**

**Variable**	**%Anaemic (total)**	**Pearson chi-square (P-value)**
**Sex**		2.45 (0.117)
Male	64.33 (2,092)	
Female	62.83 (2,085)	
**Residence**		17.50 (0.000*)
Urban	53.15 (402)	
Rural	65.35 (3775)	
**Wealth**		49.07 (0.000*)
Poorest	70.79 (829)	
Poor	65.77 (964)	
Rich	66.55 (973)	
Richer	61.729 (813)	
Richest	51.88 (598)	
**Mother education**		20.31 (0.000*)
No education	67.07 (714)	
Primary	64.17 (2,917)	
Secondary	56.1 (535)	
higher	79.03 (11)	
**House hold size**		0.68 (0.410)
≤5	64.42 (1,892)	
>5	62.81 (2,285)	
**Fever**		60.31 (0.000*)
No fever	59.39 (2,738)	
With fever	71.58 (1,431)	
**Ate meat**		0.04 (0.845)
No	67.33 (2,666)	
Yes	71.45 ( 465)	
**Cough**		9.68 (0.002*)
No cough	61.61 (2,974)	
With cough	69.06 (1,171)	
**VitaminA**		7.24 (0.007*)
No	66.46 (527)	
Yes received	63.05 (3,643)	
**Stunting**		21.55 (0.000*)
No	60.5 (2,256)	
Yes	67.02 (1,714)	
**Wasting**		16.63 ( 0.000*)
No	62.7 (3,819)	
Yes	79.99 (152)	
**Underweight**		18.40 (0.000*)
No	61.85 (3,232)	
yes	69.93 (738)	
**Mother anaemia**		34.35 (0.000*)
No	62.09 (3,510)	
Yes	73.11 (577)	
**Birth Order**		1.84 (0.606)
1	64.25 (769)	
2-3	62.84 (1,504)	
4-5	62.49 (1,058)	
6+	65.72 (846)	

The Pearson chi-square p-value with* indicate that the variable was significant at 5% significance level.

### Empirical Bayesian results

#### Model selection

The choice of the better model is based on Alkaike Information Creterion(AIC) and the Generalized Cross Validation(GCV) as used by [32] when they used empirical Bayesian method in estimation of the STAR model. A model with the smallest AIC and GCV is considered as a better model. The AIC and GCV (Table 3) favours the geo-additive model, that is, Model 4, since it has the smallest AIC and GCV. Discussion of the results will therefore be based on Model 4, the geo-additive model.

**Table 3:** **Summary of four binary logistic models**

**Variable**	**Model 1**	**Model 2**	**Model 3**	**Model 4**
	**Coeffecient**	**Coeffecient**	**Coeffecient**	**Coeffecient**
	**(95% CI)**	**(95% CI)**	**(95% CI)**	**(95% CI)**
**Constant**	2.236^*^ (1.730 2.742)	0.781^*^ (0.454 1.107)	2.081^*^ (1.495 2.666)	0.597^*^ (0.164 1.030)
**Residence**				
Rural	_	_	_	_
Urban	−0.206 (−0.465 0.053)	−0.193 (−0.453 0.068)	−0.118 (−0.395 0.158)	−0.104 (−0.381 0.173)
**Child sex**				
Female	_	_	_	_
Male	0.068 (−0.075 0.211)	0.066 (−0.077 0.209)	0.085 (−0.061 0.228)	0.080 (−0.065 0.224)
**Mother education**				
No education	_	_	_	_
Primary	−0.275^*^ (−0.486 −0.065)	−0.275^*^ (−0.485 −0.065)	−0.116 (−0.334 0.101)	−0.118 (−0.335 0.099)
Secondary	−0.372^*^ (−0.667 −0.076)	−0.369^*^ (−0.666 −0.072)	−0.190 (−0.493 0.113)	−0.192 (−0.497 0.112)
Higher	0.258 (−1.124 1.640)	0.254 (−1.135 1.642)	0.453 (−0.910 1.817)	0.436 (−0.936 1.808)
**Wealth index**				
Poorest	_	_	_	_
Poor	−0.154 (−0.380 0.071)	−0.147 (−0.373 0.079)	−0.153 (−0.382 0.076)	−0.148 (−0.377 0.082)
Rich	−0.137 (−0.364 0.090)	−0.139 (−0.366 0.089)	−0.122 (−0.353 0.110)	−0.122 (−0.355 0.110)
Richer	−0.223 (−0.459 0.014)	−0.215 (−0.452 0.021)	−0.207 (−0.449 0.035)	−0.199 (−0.442 0.043)
Richest	−0.475^*^ (−0.753 −0.197)	−0.468^*^ (−0.747 −0.189)	−0.489^*^ (−0.775 −0.203)	−0.481^*^ (−0.768 −0.194)
**Fever**				
No	_	_	_	_
Yes	0.454^*^ (0.286 0.621)	0.449^*^ (0.281 0.617)	0.448^*^ (0.277 0.620)	0.442^*^ (0.270 0.614)
**Cough**				
No	_	_	_	_
Yes	−0.013 (−0.187 0.161)	−0.019 (−0.194 0.156)	0.035 (−0.143 0.213)	0.028 (−0.151 0.207)
**Vitamin A**				
No	_	_	_	_
Yes	−0.154 (−0.377 0.069)	−0.117 (−0.341 0.107)	−0.118 (−0.346 0.110)	−0.082 (−0.311 0.147)
**Stunting**				
No	_	_	_	_
Yes	0.265^*^ (0.107 0.422)	0.288^*^ (0.129 0.446)	0.291^*^ (0.131 0.450)	0.314^*^ (0.153 0.474)
**Underweight**				
No	_	_	_	_
Yes	0.108 (−0.103 0.318)	0.100 (−0.110 0.310)	0.102 (−0.111 0.315)	0.098 (−0.115 0.311)
**Wasting**				
No	_	_	_	_
Yes	0.331 (−0.107 0.768)	0.324 (−0.116 0.764)	0.315 (−0.128 0.759)	0.309 (−0.136 0.755)
**Mother anaemia**				
No	_	_	_	_
Yes	0.681^*^ (0.461 0.901)	0.686^*^ (0.466 0.906)	0.601^*^ (0.377 0.824)	0.605^*^ (0.382 0.828)
**Child age**	−0.034^*^ (−0.040 −0.029)		−0.035^*^ (−0.041 −0.029)	
**Months of breast feeding**	−0.012 (−0.025 0.000)		−0.013 (−0.025 −0.001)	
**Mother age**	−0.004 (−0.015 0.008)		−0.003 (−0.015 0.008)	
**Variance Components**				
**Spatial effect**			0.6348	2.275
**Non-linear effects**				
Child age ${\tau}_c^2$		0.115		0.119
Mother age ${\tau}_m^2$		0.128		0.147
Breast Feeding ${\tau}_{bf}^2$		0.000		0.000
**Model fit**				
AIC	4461.33	4443.66	4402.42	4386.81
GCV	1.20699	1.20258	1.18882	1.18489

Model 1(fixed effects), Model 2(fixed plus non linear effects), Model 3(fixed and spatial effects), and Model 4(geo-additive). _ means reference category, and ^*^ means significant at 5% significance level.

#### Fixed effects

Fixed effects variables found to be significant to childhood anaemia (Table 3) are fever, wealth family of richest category, stunting and mother anaemia status. The coefficient for fever is positive which means children who have fever have increased risk to childhood anaemia compared to children who have no fever. Children of richest family have reduced risk to childhood anaemia than those who belong to poorest family, since the coefficient for the richest family is negative. Coefficient for stunting is positive, which means stunted children have a higher risk of childhood anaemia compared to children who are not stunted. Mother anaemia status has a positive effect to childhood anaemia, that is, children of anaemic mothers have their risk to childhood anaemia more than children whose mothers are not anaemic.

#### Non linear effects

**Figure 1:**
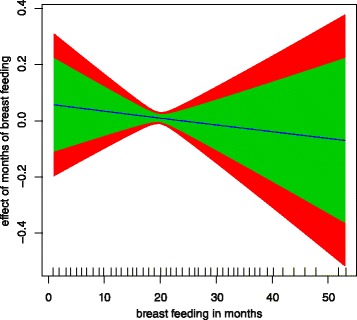
**Non linear effect of months of breast feeding to childhood anaemia.** Green band (80% CI), and red (95% CI).

Months of breast feeding has an insignificant non linear effect to childhood anaemia (Figure 1) since the variance parameter for the effect of months of breast feeding is zero (Table 3) which means assumption of non linearity does not hold.

As a matter of fact the effect of months of breast feeding is linear with childhood anaemia decreasing as months of breast feeding increases.

**Figure 2:**
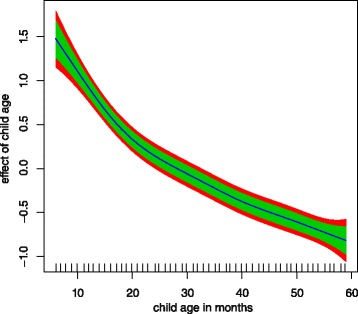
**Non linear effect of child age to childhood anaemia.** Green band (80% CI), and red (95% CI).

Child age has somewhat significant non linear effect to childhood anaemia (Figure 2) since the variance parameter for the effect of child age is not zero (Table 3). As child age increases, its effect on child anaemia decreases, that is, older children are less likely to have childhood anaemia. The chance of having anaemia is much higher in children aged about 6 months to about 20 months and decreases there after.

**Figure 3:**
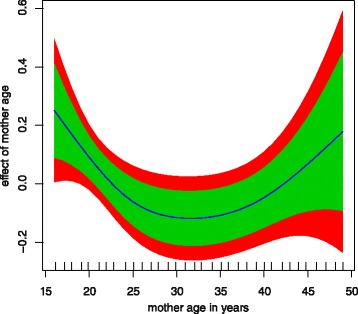
**Non linear effect of mother age to childhood anaemia.** Green band (80% CI), and red (95% CI).

Mother age has a significant non linear effect to childhood anaemia (Figure 3) since the variance parameter for its effect is not zero (Table 3). There is a U functional relationship between childhood anaemia and mother age. Young mothers are more likely to have children who are anaemic; in particular mothers aged 15 years to about 25 years. The risk to childhood anaemia remains reduced for mothers aged 22 to about 40 years. Childhood anaemia risk then rises for mothers who are aged 40 years and above.

#### Spatial effects

**Figure 4:**
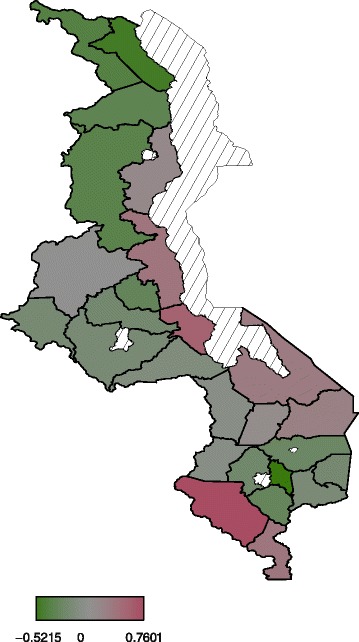
**Residual spatial effects to childhood anaemia.**

**Figure 5:**
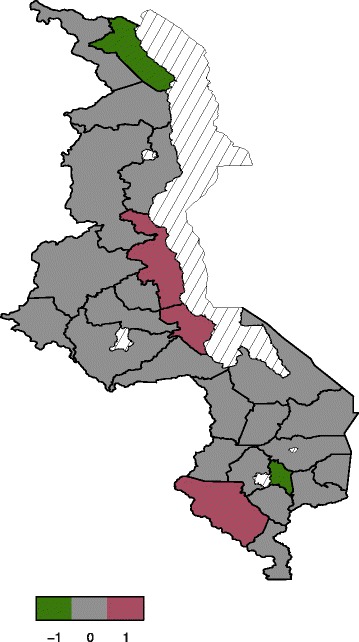
**The 95% posterior credible intervals map.** Green (negative effect), gray (insignificant effect) and red (positive effect).

**Figure 6:**
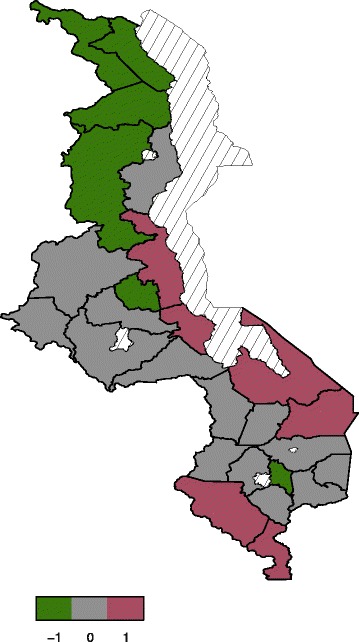
**The 80% posterior credible intervals map.** Green (negative effect),gray (insignificant effect) and red (positive effect).

Spatial effects are surrogates of unknown influences, for example climatic and environmental factors, access to good transport system, and access to good child health care services. These unknown factors may have a localized effect or global effect. Figure 4 presents total residual spatial effects to childhood anaemia. There is evidence of residual spatial effects to childhood anaemia in Malawi with Chikwawa, Nkhota-kota and Salima showing significant positive effects while Karonga and Chiradzulu show negative effects with regard to the 95% posterior credible intervals map (Figure 5). For the 80% posterior credible intervals map, Nkhota-kota, Salima, Chikhwawa, Nsanje, Mangochi and Machinga have significant positive effects while Karonga, Chitipa, Rumphi, Mzimba, Ntchisi, and Chiradzulu have significant negative effects (Figure 6).

## Discussion

This study employed the use of geo-additive logistic model to study the relationship between childhood anaemia and its risk factors. The geo-additive model allowed the mapping of residual spatial effects to childhood anaemia while accounting for non-linear covariate effects under the assumption of additiviness. Modelling of metrical continuous covariates non linearly revealed their subtle influences that could not be observed when modelled linearly. The incorporation of spatial effect in the models made some covariates not to be significant anymore. For example, mother education primary and secondary level coefficients were found to be significant in Model 1 and Model 2 (Table 3) where there was no spatial effect, but were not significant in Model 3 and Model 4 (Table 3) when the spatial effect was included in the models. Actually, the spatial component in Model 3 and Model 4 according to [28] helped to avoid underestimate model parameter standard errors which could result in significance of the covariates.

The observed residual spatial pattern in childhood anaemia shows most districts in the north reducing child anaemia, and the districts that increased risk of anaemia were all close to water bodies. The observed spatial heterogeneity may be due to unobserved factors not captured by the covariates in the models, and it is a matter of conjecture to identify them. Geographical difference in anaemia-causing infections, like malaria, hook worms and helminths could be one cause of such spatial variation. Malaria is common in places close to water bodies and where temperatures are high (above 21%). According to [33], the optimum temperature for mosquitoes development is between 22 and 32°C. Similarly, soil moisture and relative atmospheric humidity are also known to influence the development and survival of ova and larvae for hookworms and helminths, where higher humidity is associated with faster development of ova [34,35]. Salima, Nkhota-kota, Mangochi, Machinga showed positive spatial effect to anaemia at 20% significance level probably due to lake Malawi, Lake Malombe, Lake Chiuta and Lake Chilwa, and Shire River which enhance the development of mosquitoes, hookworms and helminths. Transmission of hookworms and helminths along such water bodies would also be facilitated by open faecal disposal according to [36], since along these water bodies, open faecal disposal is common particularly by fisher men. Similarly, Nsanje and Chikhwawa districts had a positive effect to child anaemia probably because they are characterised by permanent wetlands (Ndindi and Elephant marsh) with large stretches of stagnant water, and that their temperatures are above 21°C which provide the best ground for the mosquitoes to breed, resulting in increased malarial transmission and let alone malaria anaemia.

Altitude difference is another possible cause of spatial heterogeneity in anaemia. According to [27], people residing at higher altitudes (greater than 1,000 meters (3,300 feet)) have higher Hb levels than those residing at sea level. This variation is due to the lower oxygen partial pressure at higher altitudes, a reduction in oxygen saturation of blood, and a compensatory increase in red blood cell production to ensure adequate oxygen supply to the tissues. Highland areas also have lower temperatures and thus are associated with less risk to malaria anaemia. Most areas in the north like Rumphi, Mzimba, Chitipa and part of Karonga are at high altitude, and this may explain their negative effect to anaemia. The effect of altitude on geographical variation of anaemia in this study may however be due to malaria-altitude relationship and not altitude-Hb level relationship as the later was accounted for by adjusting child Hb level for altitude according to DHS guide to statistics (see [27]).

Regional nutritional disparities may also explain the spatial heterogeneity of childhood anaemia in Malawi. The cause of regional nutritional differences can be natural disasters like floods, and varying climatic conditions. Most valleys in Malawi, notably those of the Shire and Kasitu Rivers, and the southern end of Lake Malawi, are in rain shadows. Thus high risk of child anaemia in Chikhwawa, and Nsanje district may also be explained by floods from Shire River which annually destroys crops there by affecting the general nutrition of the area. Furthermore, these districts are in the Shire River basin which is a rain shadow area.

The fixed effects factors of childhood anaemia significant in this study are fever, wealth family of richest category, stunting and mother anaemia status. The finding of fixed effects factors generally confirm with what is known in the literature. The finding of fever agrees with that of [37] where fever had a positive effect. According to [37], fever is a common symptom of acute and chronic inflammatory diseases, mostly infections, which have been associated with lower Hb levels. Existing anaemia is aggravated by underlying inflammation, which leads to alterations in iron homeostasis, impaired erythrocyte proliferation, blunted erythropoietin response, and decreased erythrocyte half-life. Moreover, several pro-inflammatory cytokines have been implicated in chronic inflammation anaemia, including interleukin- (IL-) 1b, tumour necrosis factor-a (TNF-a), and IL-6.

Child age has been found to have non linear effect. Younger children are at higher risk of childhood anaemia compared to older children. This may be explained by the high demand for iron to ensure accelerated physical growth during the first year of life, and by the difficulty mothers and guardians have ensuring adequate iron consumption after the sixth month of life, when stored iron is depleted and iron needs must be met through feeding.

Children of richest family have been found to have a reduced risk to childhood anaemia compared to the poorest children. This is probably due to good nutritious food the family affords, resulting into non anaemia. Mothers who are anaemic are also prone to have anaemic children. This finding is consistent with that of [10]. The association between child’s haemoglobin level and maternal haemoglobin level may have multiple pathways. For example, maternal anaemia during pregnancy contributes to low birth weight and premature birth, both of which increase the risk of childhood anaemia. Low birth weight has been found to be risk factor of childhood anaemia by [13]. Severe maternal anaemia may also reduce breast milk iron content which can result in childhood anaemia.

Stunting positive effect on child anaemia can be due to chronic food shortage which results in reduced haemoglobin levels. Ngnie-Teta et al. [21] found a similar positive effect of stunting on childhood moderate to severe anaemia in Benin and Mali. Breast feeding had a linear effect which is consistent with most studies like that of [11]. Less months of breast feeding is associated with slightly high risk of anaemia and more months of breast feeding with less risk. Breast milk basically is said to have iron which is used in blood formation. Mother age had a non linear effect. Increased childhood anaemia for young mothers is probably due to young mothers requiring more iron for their growth there by affecting child haemoglobin level, and also elder mothers need more iron due to old age which can also affect child haemoglobin levels.

The study was not without weaknesses. The primary limitation of this study was its cross-sectional design. Despite the robustness of the analyses, control for the principal confounders, and the consistency of the main results with those of other studies on anaemia, no causal inference can be made. Moreover, because the analysis was based on an existing data set, we were limited to the use of variables found in the MDHS 2010. For instance, our study did not take into account the effect of early umbilical cord clamping after birth, which several studies have considered an important anaemia determinant [38].

## Conclusion

In summary, there is evidence of residual spatial effect to childhood anaemia in Malawi. While government and non governmental organizations concerned with child health should be geared in treating childhood anaemia by focusing on known measurable factors like mother anaemia status, child age, mother age, family wealth, child fever and stunting which have been found to be significant in this study, attention should also be put to effects of unknown or unmeasured factors to childhood anaemia present at community level. Special attention to these unknown factors to childhood anaemia should be put to districts like, Nkhota-kota, Salima, Chikhwawa, Nsanje, Mangochi and Machinga that have shown significant positive spatial effects.

## Abbreviations

AIC: Alkaike information creterion
BIC: Bayesian information criterion
CI: Credible interval
EB: Empirical Bayes
GVC: Generalized cross validation
Hb: Haemoglobin
HIV: Human immune-deficiency virus
ICC: Intra-class correlation coefficient
MDHS: Malawi demographic health survey
NDVI: Normalized difference vegetation index
NSO: National statistics office
REML: Restricted maximum likelihood
SSA: Sub Saharan Africa
STAR: Structured additive regression
